# Genomic Selection in Preliminary Yield Trials in a Winter Wheat Breeding Program

**DOI:** 10.1534/g3.118.200415

**Published:** 2018-06-26

**Authors:** Vikas Belamkar, Mary J. Guttieri, Waseem Hussain, Diego Jarquín, Ibrahim El-basyoni, Jesse Poland, Aaron J. Lorenz, P. Stephen Baenziger

**Affiliations:** * Department of Agronomy and Horticulture, University of Nebraska-Lincoln, Lincoln, NE 68583; † USDA, Agricultural Research Service, Center for Grain and Animal Health Research, Hard Winter Wheat Genetics Research Unit, Manhattan, KS 66502; ‡ Crop Science Department, Faculty of Agriculture, Damanhour University, Egypt; § Wheat Genetics Resource Center, Department of Plant Pathology, Kansas State University, Manhattan, KS 66506; ** Department of Agronomy and Plant Genetics, University of Minnesota, St. Paul, MN 55108

**Keywords:** genomic prediction, *Triticum aestivum*, spatial variation, genotyping-by-sequencing, genomic best linear unbiased prediction, Genomic selection, shared data resources, GenPred

## Abstract

Genomic prediction (GP) is now routinely performed in crop plants to predict unobserved phenotypes. The use of predicted phenotypes to make selections is an active area of research. Here, we evaluate GP for predicting grain yield and compare genomic and phenotypic selection by tracking lines advanced. We examined four independent nurseries of F_3:6_ and F_3:7_ lines trialed at 6 to 10 locations each year. Yield was analyzed using mixed models that accounted for experimental design and spatial variations. Genotype-by-sequencing provided nearly 27,000 high-quality SNPs. Average genomic predictive ability, estimated for each year by randomly masking lines as missing in steps of 10% from 10 to 90%, and using the remaining lines from the same year as well as lines from other years in a training set, ranged from 0.23 to 0.55. The predictive ability estimated for a new year using the other years ranged from 0.17 to 0.28. Further, we tracked lines advanced based on phenotype from each of the four F_3:6_ nurseries. Lines with both above average genomic estimated breeding value (GEBV) and phenotypic value (BLUP) were retained for more years compared to lines with either above average GEBV or BLUP alone. The number of lines selected for advancement was substantially greater when predictions were made with 50% of the lines from the testing year added to the training set. Hence, evaluation of only 50% of the lines yearly seems possible. This study provides insights to assess and integrate genomic selection in breeding programs of autogamous crops.

Wheat (*Triticum aestivum*) is a cereal crop with the highest hectarage in the world. In contrast to maize (*Zea mays*) and soybean (*Glycine max*), wheat varieties developed by the public sector account for a majority (∼75–90%) of wheat acreage in the United States. Public sector breeding programs previously relied primarily on phenotypic data or a combination of phenotype and molecular markers associated with traits controlled by one or a few genes to make selections (Bernardo 2016; Randhawa *et al.* 2013). With the rapid decline in sequencing costs and the ease of generating genome-wide markers with genotyping methods such as genotyping-by-sequencing (GBS; Poland and Rife 2012), genomic selection (GS) has become a powerful tool to enhance selection for quantitative traits and consequently increase genetic gain over time (Desta and Ortiz 2014; Jarquín *et al.* 2017; Pérez-Rodríguez *et al.* 2017; Poland 2015; Poland and Rife 2012; Spindel and McCouch 2016; Yu *et al.* 2016). There is now increased interest in integrating GS into public sector breeding programs.

Recent literature on genomic prediction (GP) has focused on estimating predictive ability (PA) of various traits using different GP models and cross-validation schemes. Arruda *et al.* (2016) and Kooke *et al.* (2016) compared the genome-wide association studies and GP using cross-validations to determine the PA for *Fusarium* head blight resistance in wheat and various morphological traits in Arabidopsis (*Arabadopsis thaliana*). Similarly, Velu *et al.* (2016) investigated the PA through cross-validations for grain zinc and iron concentrations in spring wheat. These studies conclude that GS holds promise for improving the respective traits. The testing of various GP models to obtain increased PA is an active area of research. Linear models such as genomic best linear unbiased prediction (GBLUP) utilize all of the markers genotyped for construction of the genomic relationship matrix (GRM), whereas Bayesian variable selection models assume that a reduced number of markers explain the genetic variance of a trait. Nonlinear models such as reproducing kernel Hilbert space and neural networks are also used for GP. Genomic prediction models and their assumptions and features have been previously reviewed in other articles (Desta and Ortiz 2014; Heslot *et al.* 2012; Howard *et al.* 2014).

Although testing PA is critical information for GS, a large gap exists between findings in these studies and their application in breeding programs (Bernardo 2016). A limited number of articles have described the utilization of GS in real case scenarios such as for germplasm or cultivar development (Asoro *et al.* 2013; Bernardo 2016; Beyene *et al.* 2015; Combs and Bernardo 2013; Massman *et al.* 2013; Rutkoski *et al.* 2015). Tracking of lines advanced in a plant breeding program and comparing their observed and predicted phenotypes can provide insights to researchers for using GS in the breeding program. In this study, we test the performance of GP to predict grain yield and evaluate how GS can be used for making selections in preliminary yield trials, one of the critical stages in the University of Nebraska winter wheat breeding program (Figure S1 in File S1).

The University of Nebraska winter wheat breeding program makes over 1,000 unique crosses annually and the progenies from these crosses are tested systematically over the subsequent 11 years to identify a cultivar for release (Figure S1 in File S1; Baenziger *et al.* 2011; Baenziger *et al.* 2001). The F_2_ and F_3_ nurseries are grown in bulk populations and nearly 45,000 heads selected from the F_3_ bulks are planted as headrows (F_3:4_). The selections made in F_2_, F_3_, and F_3:4_ nurseries are primarily based on disease resistance, winterhardiness, and plant type. Experimental lines are evaluated for yield along with other traits such as agronomic performance and end-use quality starting from F_3:5_ onwards. The observation yield trial (OYT; F_3:5_ nursery) is grown in plots at one location for grain yield and in rows at a second location for winterhardiness and disease resistance due to the limited amount of seed. The preliminary yield trial (PYT; F_3:6_ nursery), the first multi-location yield trial, is then grown in one or two replicates as augmented trials with replicated check cultivars. The advanced yield trial (AYT; F_3:7_ nursery) and elite yield trials (EYTs, F_3:8_ to F_3:12_ nurseries) are grown in a replicated alpha lattice design in multiple locations, and lines are tested for more than one year. Subsequently, a few elite lines are advanced to regional nurseries such as Southern Regional Performance Nursery (SRPN), state cultivar testing, and foundation seed increase. The SRPN are replicated trials comprising 50 entries contributed by various breeding programs in the Great Plains and is grown at more than 30 locations, with sites in Texas, Oklahoma, Kansas, Colorado, Nebraska, and South Dakota. The SRPN trials are coordinated by the USDA-ARS at Lincoln, NE. Phenotypic selection for yield is most accurate in the AYT and EYTs due to the extensive replications and testing of the lines at multiple environments for multiple years. However, in the early generation (mainly F_3:5_) and PYT, phenotypic selection for yield is less effective either due to the absence of replication or testing done primarily in few environments, which do not represent the diverse range of environments possible when wheat is grown in Nebraska. The inaccuracy of phenotypic selection for yield in trials can be overcome or at least reduced by using GS, which has the potential to increase selection accuracy over phenotypic selection.

In the present study, we focus on the PYT, from which lines are advanced to enter the resource demanding AYT and EYTs, grown with replication at multiple locations (Figure S1 in the File S1). Hence, making accurate selections in the PYT is important to advance lines that have the greatest expectation to subsequently perform well. In addition, selection of top-performing lines from the PYT allows for recycling of elite lines sooner to the subsequent breeding cycle as parents before testing them in the AYT and EYTs. Increasing selection accuracy and reducing the breeding cycle time will increase genetic gain over time (Bassi *et al.* 2016). The specific objectives of this study were to: (1) determine how GP can predict grain yield of new lines (non-phenotyped) in the PYT and assist with selections; (2) compare GS and phenotypic selection for making advancement from PYT to AYT; and (3) compare genomic estimated breeding values (GEBVs) and phenotypic estimates of lines advanced from the PYT to the AYT and EYTs to determine the use of GS for making selections in the breeding program. It should be noted that although selections made from PYT (F_3:6_) onwards take into account agronomic performance, end-use quality, and disease resistance among other factors in the breeding program, yield is the most important trait considered for selections (Baenziger *et al.* 2011; Baenziger *et al.* 2001).

## Materials and Methods

### Plant material

Four independent PYT nurseries (F_3:6_) of the University of Nebraska winter wheat breeding program grown in 2012-2015 were utilized for GP (File S2). Each year ∼270 lines are tested in the PYT nursery. Two hundred and eighty experimental lines were grown in 2012 and 2013 and 270 experimental lines in 2014 and 2015 thus a total of 1,100 unique lines were used in this study. There was no overlap between experimental lines tested across years. The experimental design was an augmented design with 10 incomplete blocks. Each incomplete block in 2012 and 2013 had 28 lines and two different check cultivars and in 2014 and 2015 there were 27 lines and three different check cultivars. Thus, 270 or 280 lines along with 20 or 30 check plots (each check cultivar was replicated 10 times) were grown in a single trial. Trials were grown as a single replicate, or sometimes two replicates, at 8 to 10 locations annually (File S2). Testing locations include Mead, Lincoln, Clay Center, North Platte, McCook, Grant, Sidney, and Alliance in Nebraska, and Hutchinson and Mount Hope in Kansas (File S2). Yield measured on the lines in the PYT nurseries (33 environments) was used for GS.

We utilized four AYT nurseries (F_3:7_) grown in 2013-2016 to test the effectiveness of GS for making selections using GEBVs for yield from the PYT nurseries grown in 2012-2015. The AYT nursery was grown in an alpha lattice experimental design with 57 lines and three different check cultivars each year and had either two or three replicates at each location (File S2). The 57 lines in AYT are selected from PYT primarily based on yield, and also end-use quality and disease resistance, and represent ∼21% of top performing lines in the PYT. There was no overlap between the lines tested across years and a total of 228 unique lines were tested across four years. Each year the nursery was tested at six to nine locations with a total of 29 environments across four years. The locations were the same Nebraska locations as described earlier for the PYT nursery (File S2).

Experimental lines in both the PYT and AYT nurseries were grown in plots of four rows of 3.0 m length and 0.30 m spacing between the rows. Each plot was planted at a seeding rate of 54 kg ha^-1^. Yield was measured from a combine harvest of all four rows of each plot.

### Phenotypic data analysis

Grain yield at each location within a year was analyzed separately to account for spatial variation in the field and obtain the best estimates of the phenotype. Each PYT nursery was analyzed using mixed models that accounted for either experimental design features such as incomplete blocks, rows, and columns or spatial variation in the field (Table S1 in File S1; Gilmour *et al.* 1997). Model performance was assessed using Akaike Information Criterion (AIC; Piepho and Williams 2010; Wolfinger 1993) with all terms except checks fit as random effects (Table S1 in File S1). Histogram, quantile-quantile plot, and residual plots were used to inspect normality of residuals, heteroscedasticity, and outliers in the dataset. If a mixed model accounting for spatial variation had better performance (smaller AIC values, and normal distribution and reduced heteroscedasticity of residuals) compared to models that accounted for just the experimental design features, we tested additional models that utilized the best spatial variation adjustment plus the experimental design features (Table S1 in File S1; Gilmour *et al.* 1997). Finally, the mixed model that performed best was used to generate best linear unbiased predictors (BLUPs) for each line at each location. Subsequently, the lines grown at different locations within a year were treated as replicates and BLUPs derived for each line at each location within a year were averaged into a single BLUP value for each line. This is a reasonable approach as all lines in PYT are tested in all locations and the dataset is well balanced. Besides this approach, there are many alternative ways to analyze multi-environment trials. For instance, models that account for covariance structure between environments, heterogeneity of residuals, and deregressing BLUPs following a one-step analysis (Cullis *et al.* 1998; Garrick *et al.* 2009; Piepho *et al.* 2012; Welham Sue *et al.* 2010).

The AYT nursery was also analyzed separately at each location within a year and one linear model was used to generate the BLUP value for each line at each location. The model used to analyze yield data of the AYT nursery is as follows:

y=μ+r+b(r)+g+e (1)

where y is the vector of phenotypes, μ is the mean, r is the replicate, b(*r*) is the incomplete block nested within a replicate, g is the line effect, and e is the error term. Replicate, block and lines were treated as random effects. Analyzing the PYT and AYT nursery separately at each location was necessary to test and account for the spatial variation at each location or inspect the quality of the yield data from each of the location before combining them into a single BLUP value for GP analysis.

Broad-sense heritability (H) was estimated for the PYT nurseries at each of the locations grown in two replicates (File S2). The PYT nurseries that were not replicated, but had two to three different check cultivars grown in each of the augmented trials, separated the genetic and residual variances and were used to calculate the H. The variance components were generated using the mixed model that performed the best for each location. The H was also estimated at each of the locations for the AYT nurseries (File S2). The variance components were generated using the equation (1) and the H was estimated using the following equation:

$$H=\sigma_{G}^{2}/(\sigma_{G}^{2}+\frac{\sigma_{R}^{2}}{r})$$ (2)

where $\sigma_{G}^{2}$ and $\sigma_{R}^{2}$ are the variance of the lines and the residuals and *r* is the number of replicates within the location. Heritability was estimated across locations in both the PYT and the AYT nurseries (He *et al.* 2016). Variance components were generated using the linear model:

y=μ+L+g+e (3)

where y is the vector of BLUPs estimated at each of the locations within a year, μ is the mean, L is the location effect, g is the line effect, and e is the error term. The location was set as a fixed effect because the locations were preselected in the breeding program and the line effect was set as a random effect. The H was estimated using the following equation:

$$H=\sigma_{G}^{2}/(\sigma_{G}^{2}+\frac{\sigma_{R}^{2}}{L})$$ (4)

where $\sigma_{G}^{2}$ and $\sigma_{R}^{2}$ are the variance components of the line and the residuals and L is the number of locations tested within a year (He *et al.* 2016).

All models were fit using ASreml v3.0 (Gilmour *et al.* 2009) in the R programming environment (R Core Team 2016). The R script and an example dataset are provided (Files S3, S4).

### DNA extraction and genotyping-by-sequencing

Five seeds of each line in the PYT nursery were grown annually in the University of Nebraska-Lincoln (UNL) greenhouse and young leaves were bulked and harvested from 2-week old seedlings in 96-well plates. Two randomly chosen wells were kept blank in each plate for quality control purposes. The leaves were dried and desiccated by placing the plates in boxes filled with silica beads. DNA was extracted using BioSprint 96 DNA Plant Kit from QIAGEN per the manufacturer’s protocol. The genotyping-by-sequencing (GBS) was performed at the Wheat Genetics and Germplasm Improvement Laboratory (WGGIL) at Kansas State University. Samples were quantified using PicoGreen and GBS libraries constructed in 188-plex using the P384A adaptor set (Poland *et al.* 2012b). DNA was co-digested with two restriction enzymes, a rare-cutter *PstI* (CTGCAG), and a frequent-cutter *MspI* (CCGG), and barcoded adapters were ligated to individual samples. The GBS library was prepared by pooling samples from two 96 well plates and amplified in a thermocycler. Each library was then sequenced in a single flow cell lane of Illumina HiSeq. Detailed protocols and updates on the GBS protocol can be found on the WGGIL website (http://www.wheatgenetics.org).

### SNP calling, quality control, anchoring to the genome, and imputations

Quality inspection of sequence files and SNP calling was performed on the high-performance computing clusters available at the Holland Computing Center (http://hcc.unl.edu) at UNL. The sequence quality was inspected using FASTQC (http://www.bioinformatics.babraham.ac.uk/projects/fastqc/). SNP calling was done using the TASSEL GBS pipeline v4.0 (Glaubitz *et al.* 2014) and merging of multiple samples of the same line was done in version 3. SNP calling was implemented using default parameters except for the criterion of a number of times a GBS tag to be present to be included for SNP calling was changed from the default value of 1 to 5 to increase the stringency in SNP calling. A pseudo-reference genome built using the International Wheat Genome Sequencing Consortium Chromosome Survey Sequences (CSS) v2.0 of the hexaploid bread wheat variety *Chinese Spring* was utilized for SNP calling (International Wheat Genome Sequencing Consortium (IWGSC) 2014). The GBS data of 1,100 lines of the four PYT nurseries grown in 2012-2015 were analyzed along with an additional 2,202 lines to get a better coverage of the genome, increasing read depth, and for inspecting SNP calling accuracy (Zhang *et al.* 2015). These additional samples included one bi-parental mapping population, an F_3:5_ nursery of the breeding program grown in 2015, and check cultivars. A total of 3,302 unique samples were used for SNP calling. We compared SNP calls of 20 lines genotyped by GBS at least twice (biological replicates) over the years for assessing the SNP calling accuracy (File S5). SNP markers with missing information in more than 80% (Torkamaneh and Belzile 2015) of the samples were excluded using VCFtools (–max-missing 0.20; Danecek *et al.* 2011) and the distribution of missing values across SNPs and lines was investigated (Figure S2 in File S1). Imputations were performed on the remaining set of markers in Beagle v4.0 (Browning and Browning 2007). Further, SNP markers with minor allele frequency less than 0.05 (–maf 0.05) and allelic R^2^ estimated using Beagle less than 0.5 (SNPs that could be difficult to impute accurately) were excluded (Figure S2 in File S1; Browning and Browning 2007). The genomic location information of the SNPs was derived using the positions of the CSS contigs that were anchored and ordered by population sequencing and a high-density linkage map (Chapman *et al.* 2015).

### Genomic prediction and cross-validation design

Phenotypic response of the line ($y_{i}$) can be described as the sum of an overall mean (μ), plus the random effect of the line ($L_{i}$), plus an error term ($e_{ij}$) as follows:

$$y_{ij}=\mu +L_{i}+e_{ij}$$ (5)

where line effects are IID (independent and identically distributed) drawn from a normal distribution of the form $L_{i}∼N\left(0,I\sigma_{L}^{2}\right)$, $L_{i};i=1,\ldots ,I$, and $e_{ij}∼N\left(0,I\sigma_{e}^{2}\right)$. In equation (5) the effects of the different levels of random line effect are independent and the information is not borrowed across lines. When genome-wide markers are available, the genomic values of all the lines are normally distributed with $\text{g}∼N\left(0,\text{G}\sigma_{\text{g}}^{2}\right)$, where G is the GRM calculated using genome-wide SNP markers and $\sigma_{\text{g}}^{2}$ is the genomic variance. Collecting the aforementioned assumptions the final GBLUP model results as:

$$y_{ij}=\mu +g_{i}+e_{ij}$$ (6)

where $y_{ij}$ is the vector of phenotype (for example, BLUPs of lines grown in 4 years), and $g∼N\left(0,\text{G}\sigma_{\text{g}}^{2}\right)$ and $e∼N\left(0,I\sigma_{e}^{2}\right)$. In equation (6) the effects of the different levels of random line effect are correlated as per the off-diagonal values of GRM and there is potentially borrowing of information across lines, which allows for predicting the phenotype of lines that are missing.

The GRM was estimated as in Perez and de los Campos (2014). Briefly, the SNP markers obtained after quality control were converted from nucleotide format to numerical format using the *NumericalGenotypePlugin* implemented in the TASSEL software (Bradbury *et al.* 2007). Genotype matrix (X) was multiplied by 2 to change the genotype scale to 2, 0, and 1 for homozygous major, homozygous minor, and heterozygous before estimating GRM. The X matrix was centered and scaled by subtracting each value in the column from column means and by dividing with their standard deviations using the *scale* function in R. Subsequently, GRM was estimated as:

$$GRM=\frac{XX^{\prime }}{ncol(X)}$$

where ncol(X) is the number of SNP markers.

The BGLR function in the Bayesian Generalized Linear Regression (BGLR; Perez and de los Campos 2014) package in R was used to fit the GBLUP model and genomic estimated breeding values were obtained. Each run was performed with 12,000 iterations and 2,000 burn-in. The BGLR function with default values was used for assigning prior distribution and hyper-parameter settings (de los Campos *et al.* 2013; Perez and de los Campos 2014).

The cross-validation scheme was designed to investigate how GP can predict grain yield of PYT and address the practical scenarios that may occur in a breeding program (Table 1). For example, an occurrence of extreme weather events such as hailstorms during a breeding season may result in loss of a subset of the trial. The rest of the lines within the same location are not damaged, and the data from the undamaged lines will be available to combine with the training set and predict the phenotype of damaged lines. Another example where the cross-validation scheme will have practical utility is to use GS as a tool for allocation of breeding program resources. If we determine the number of lines that can be predicted with reasonable accuracy, it may be possible to grow only a subset of lines in the trial and predict the rest and save costs by reduced phenotyping. In our cross-validation scheme, the training set comprised F_3:6_ lines from three years and randomly chosen lines in steps of 10% from the fourth year and test set included the rest of the lines from the fourth year (Table 1). For instance, the training set may include all lines tested in 2013, 2014, and 2015 PYT nurseries, and 90% of the lines from 2012 PYT nursery randomly selected, and the test set includes the remaining 10% of the lines grown in 2012. This process was repeated 10 times by randomly sampling 90% of the lines in each run. We refer to this cross-validation scenario where 10% of the lines are excluded as NA10. Similarly, in 2012, NA50 training set will comprise all lines tested in 2013, 2014, and 2015, and 50% of the lines randomly chosen from 2012, and the test set will include the remaining 50% of the lines tested in 2012. We repeated this process 10 times by randomly selecting 50% of the lines from 2012 in each run. This cross-validation strategy was tested for each of the four years (2012-2015) with NA10 to the NA90 scenario and by randomly selecting lines 10 times (Table 1).

**Table 1:** Description of prediction scenarios and composition of training and test set using 2012 as an example

Prediction scenario	Cross-validation scheme	Training set	Test set	Size of training set	Size of test set
Predictions of 2012 F_3:6_ nursery	NA10	2013+2014+2015+90% of the lines in 2012	Rest 10% of the lines in 2012	1,072	28
	NA20	2013+2014+2015+80% of the lines in 2012	Rest 20% of the lines in 2012	1,044	56
	NA50	2013+2014+2015+50% of the lines in 2012	Rest 50% of the lines in 2012	960	140
	NA100	2013+2014+2015	100% of the lines in 2012	820	280
Predicting 2012 F_3:6_ lines advanced to F_3:7_ in 2013	—	2013+2014+2015	100% of the lines in 2012 (subset GEBV of 2012 57 F_3:6_ lines advanced to F_3:7_ in 2013)	820	280
Predicting 2012 F_3:6_ lines advanced to F_3:7_ in 2013 skipping the year of F_3:7_ (2013) in training set	—	2014+2015	100% of the lines in 2012 and 2013 (subset GEBV of 2012 57 F_3:6_ lines advanced to F_3:7_ in 2013)	540	560
Tracking lines advanced in the breeding program from 2012 F_3:6_	—	2013+2014+2015	100% of the lines in 2012	820	280
Tracking lines advanced in the breeding program from 2012 F_3:6_ including 50% of the lines from the same nursery in training set	—	2013+2014+2015+50% of the lines in 2012	Rest 50% of the lines in 2012	960	140

We also predicted the yield of all the lines in the PYT nursery in a new year using lines tested in other years (Table 1). For example, we predicted the yield of lines grown in 2012 PYT nursery using lines tested in 2013, 2014, and 2015 PYT nurseries. We refer to it as NA100. Although we are predicting older lines (for example, 2012 PYT) using newer lines (2013, 2014, and 2015 PYT) the results will be insightful for predicting a new nursery in a new year. This is true because development of a wheat cultivar usually takes ∼12 years (Figure S1 in File S1) and elite lines are recycled as parents only six to seven years after the first cross is made.

The PA (Faville *et al.* 2018; Sallam *et al.* 2015) for each run was assessed using the Pearson’s correlation between the predicted genotypic effects (GEBVs) and the adjusted observed phenotypes (BLUPs). We also calculated an average across the 10 runs to estimate average PA for each validation scenario.

### Effectiveness of phenotypic and genomic selection to make selections

The 57 lines of the ∼270 lines advanced from the PYT nursery (2012-2015) to the AYT nursery (2013-2016) were used to evaluate GS and phenotype to make selections (Table 1). The GEBVs for the 57 lines were subdivided from the GEBVs estimated for all ∼270 lines in the PYT nursery (NA100 scenario of predictions). The GS ability was evaluated by estimating the correlation between GEBV of the PYT nursery and their BLUPs in the AYT nursery. Similarly, the phenotypic selection ability was estimated by calculating the correlation between BLUPs of the PYT nursery and their BLUPs in the AYT nursery. Comparing the correlation coefficients of GS and phenotypic selection abilities would indicate the effectiveness of using phenotypic selection and GS for making selections from PYT to AYT in each of the four years. Since the PYT conducted in validation year is included in the training set, we have observed the environment where the predictions will be tested in the training set. Therefore, the predictions are effectively made for new lines and are evaluated in an observed environment. This scenario is valuable as the new growing year may experience similar environmental conditions as one of the previously observed years.

We also evaluated GS and phenotypic selection by skipping the PYT nursery tested in the AYT year in the training set (Table 1). For example, the GEBV for lines grown in 2012 PYT was estimated using lines grown in 2014 and 2015 PYT (skipping 2013 PYT) in the training set (Table 1). To match the training set comprising two PYTs to predict 2012, 2013 or 2014, the GEBVs for lines grown in 2015 are estimated using lines grown in 2012 and 2013 PYT as a training set. The downside of estimating GEBV by skipping the PYT lines from the AYT year in the training set is it reduces the size of the training set by 270 to 280 lines compared to the first approach (Table 1). A smaller population size of the training set may affect the accuracy of the GEBVs. Overall, the results from these two scenarios will allow comparison of GS and phenotypic selection for making selections.

Lastly, genomic and phenotypic selections are evaluated using the 57 lines advanced from the PYT to the AYT nursery and not all of the ∼270 lines in the PYT. These 57 lines generally represent high-yielding lines and belong to one tail of the distribution containing all lines in the PYT nursery (Figure S3 in File S1). Nevertheless, using PYT and AYT provides an opportunity to compare GS and phenotypic selection using existing datasets.

### Prospects for implementing genomic selection in the preliminary yield trial

To evaluate the prospects of implementing GS in the PYT, we tracked the lines advanced until 2016 primarily based on phenotype from the PYT nurseries grown in 2012-2015 and compared it with BLUP and GEBV values estimated on the lines in the PYT nursery. For instance, lines advanced from the 2012 PYT nursery until 2016 were selected and retained in the breeding program for five years. Similarly, lines are retained for four years starting from 2013 PYT nursery, three years from 2014, and two years from 2015. The GEBVs of the lines grown in the PYT each year were estimated using the lines from the rest of the years as training dataset (NA00 scenario), which is equivalent to predicting new lines in a new year. Further, studying the frequency of lines with above average GEBV or BLUP or both, selected from the PYT nursery together with the information on the number of years a line was retained in the breeding program will allow for evaluation of the prospects of implementing GS in the PYTs in the breeding program. We repeated this entire process by making predictions on 50% of the lines randomly selected in each year and using the remaining 50% of the lines along with the lines from other years as training dataset (NA50 scenario). The NA50 scenario is expected to have higher PA compared to NA100 scenario. Thus, this comparison using the GEBVs from NA50 scenario will test the prospects of implementing GS when PA is higher.

Phenotypic selection in the PYT nursery is primarily based on yield. Typically, lines with higher yield are selected first and then subsequently a few of these lines are dropped based on other traits such as resistance to stem rust (causal organism *Puccinia graminis* Pers.:Pers. f. sp. *tritici* Eriks. E. Henn.), agronomic performance, and end-use quality (Baenziger *et al.* 2011; Baenziger *et al.* 2001). A lower yielding line in the PYT generally does not get advanced to AYT irrespective of the values of other traits. Therefore, tracking lines advanced from the PYT based on phenotype and comparing it with BLUP and GEBV values based on yield is a relatively fair comparison to determine the prospects of implementing GS in the PYTs.

### Population structure and kinship

Population structure among the 1,110 F_3:6_ lines was investigated using principal component analysis (PCA). The PCA was performed in TASSEL (Bradbury *et al.* 2007) and a plot of PC1 *vs.* PC2 was made using the ggplot2 package (Wickham 2009) in R. The kinship between test and training set was analyzed by calculating the maximum realized kinship coefficient (MRKC; Auinger *et al.* 2016; Saatchi *et al.* 2011) using the GRM (Perez and de los Campos 2014). The MRKC was calculated as max (*U_ij_*) and *U_ij_* is the realized kinship coefficient between line *i* in the test set and line *j* in the training set. The MRKC value represents the kinship between a specific line in the test set and all lines in the training set. Further, a mean of maximum kinship coefficient values across all lines in the test set was calculated.

### Data availability

File S1 contains supplementary Tables S1-S2 and Figures S1-S7. File S2 has details of the preliminary yield trials (F_3:6_) and advanced yield trials (F_3:7_) grown in 2012-2015 and 2013-2016. The experimental design, number of locations, number of replicates per location, heritability for yield within and across locations is provided. File S3 contains the R script developed for analyzing preliminary yield trials (F_3:6_) grown in an augmented design. Mixed models incorporated both experimental design and spatial variation. File S4 provides an example phenotypic dataset for utilizing the R script provided in File S3. File S5 shows the SNP calling accuracy of 20 lines genotyped by genotyping-by-sequencing at least twice (biological replicates) over the years. The accuracy is presented as sequential pairwise comparisons of 20 unique samples. File S6 contains the yield data (best linear unbiased predictors) of the preliminary yield trials (F_3:6_) grown in 2012-2015 used for genomic selection. File S7 contains the SNP marker data of 26,925 SNPs on the 1,110 F_3:6_ lines grown in 2012-2015. The homozygous major, homozygous minor, and heterozygous genotypes are coded as 1, 0, and 0.5 in the genotype matrix. File S8 shows the Akaike Information Criterion (AIC) values and the residual plots for each of the mixed models tested for the yield data collected on the preliminary yield trial (F_3:6_) grown in McCook, NE in 2014. The rest of the analyses described in this study (except for the phenotypic data analysis conducted using ASreml v3.0) were performed using publicly available software and R packages. Supplemental material available at Figshare: https://doi.org/10.25387/g3.6249410.

## Results

### Accounting for spatial variation in field trials and broad-sense heritability

Grain yield data of the PYT nursery were analyzed using linear mixed models incorporating spatial variation. The motivation for testing and correcting for spatial variation to improve genomic predictions was derived from recent articles (Bernal-Vasquez *et al.* 2014; Lado *et al.* 2013) that showed accounting for spatial variation in the field result in either similar or higher predictive abilities. Of the 33 environments (location by year combination) tested, five environments (trials conducted by WestBred, LLC or Bayer CropScience) did not have field-layouts available and thus the model accounted for just the experimental design (incomplete block) when generating the BLUPs for the lines (Table S1 in File S1). For 27 of the remaining 28 environments, the model accounting for spatial variation showed better performance compared to models accounting for just the experimental design based on lower AIC values, normality, and reduced heteroscedasticity of residuals (Table S1 in File S1). As an example of the model selection results, a table with the AIC values and residual plots for various models is provided for the PYT nursery grown at McCook, NE in 2014 (File S8). Averaging the BLUPs of lines grown at various locations within a year significantly improved the broad-sense heritability (H). For instance, H across the PYT nurseries within a year increased from ∼0.10 (across two distant locations) to ∼0.74 (across all of the 7 to 10 locations; File S2). Higher H will result in increased predictive abilities in the GP analysis.

In the PYT nursery, the H for grain yield within a location with no replicate varied from 0.25 to 0.95, whereas at locations with two replicates H ranged from 0.62 to 0.79 (File S2). The H across locations within a year ranged from 0.63 (2013 year) to 0.81 (2012 year; File S2). In the AYT nursery, the H was generally higher than the PYT nursery with values up to 0.94 and the H across locations within a year ranged from 0.58 (2014 year) to 0.79 (2015 year; File S2). The AYT nursery’s smaller sample size (57 lines) and increased replication (two or three replicates) improved heritability relative to the PYT nursery with ∼270 lines and one or two replicates. The 2013 PYT (except at Lincoln and Clay Center) and 2014 AYT (except at Mead, Lincoln, and Clay Center) had lower heritability trials at other locations. Sequential addition of locations (starting with two geographically distant locations and then subsequently adding sites generally from the eastern to the western side of the state) within a year in the AYT nursery led to a similar increase in H as was found in the PYT nursery. The H for the AYT nursery ranged from ∼0.20 (across two distant locations) to ∼0.79 (across all of the 6 to 7 locations; File S2).

### SNP calling, quality filtering, population structure, and kinship

We performed SNP calling using the GBS data of 1,100 lines of the four PYT nurseries grown in 2012-2015 along with an additional 2,202 lines and identified 206,622 SNPs. The SNP calling accuracy tested using sequential pairwise comparisons of 20 samples genotyped using GBS at least twice was 95.7% (File S5). The 206,622 SNPs were filtered to exclude markers that had missing information in more than 80% of the samples, and this resulted in 79,118 SNPs. The distribution of missing sites across SNPs was left-skewed, and across lines, it was relatively normally distributed (Figure S2 in File S1). These SNPs were then processed using BEAGLE and the missing genotypes were imputed. The use of GBS results in low coverage and high missing information, which precludes the accurate estimation of imputation accuracy. Therefore, allelic R^2^ generated in BEAGLE was used as a filtering criterion to exclude the likely low quality imputed SNPs (Figure S2 in File S1; Browning and Browning 2007). The distribution of missing information across these selected (allelic R^2^ > 0.5) SNPs prior to imputation looks random compared to the left-skewed distribution of all SNPs used for imputation (Figure S2 in File S1). Subsequently, the imputed marker data set was subdivided to include just the 1,100 lines of the 2012-2015 PYT nurseries and filtered using two criteria: (1) MAF > 0.05; (2) MAF > 0.05 and allelic R^2^ > 0.5. Filtering using only MAF criterion resulted in 41,913 SNPs and both MAF and allelic R^2^ provided 26,925 SNPs. The Pearson’s correlation coefficient estimated between kinship coefficients estimated using 41,913 and 26,925 SNP markers was 0.99. This is not unexpected and previous studies have shown the method of imputation has little effect on the kinship and predictive abilities (Jarquín *et al.* 2014; Poland *et al.* 2012a). Hence, we decided to proceed with the higher-quality 26,925 SNPs for the subsequent analysis (File S7).

The 26,925 SNPs were distributed on all 21 chromosomes. The number of markers on a chromosome ranged from 114 (4D) to 3,323 (3B) with an average of 1,282 per chromosome (Figure S4a in File S1). Nearly 19,707 of the 26,925 SNPs were placed in CSS contigs anchored and ordered on chromosomes using population sequencing (Figure S4b in File S1). The distribution of CSS contigs containing the 19,707 SNPs across each chromosome showed good coverage of the wheat genome except for a few D genome chromosomes, previously reported to be less polymorphic (Lado *et al.* 2013). The population structure evaluated using PCA by plotting PC1 *vs.* PC2 did not indicate the presence of prominent clusters suggesting the absence of strong population structure in the PYT nurseries grown in 2012-2015 (Figure S5 in File S1). The PC1 and PC2 explained 4.3% and 3.2% of the variation in the dataset (Figure S5 in File S1).

**Figure 1:**
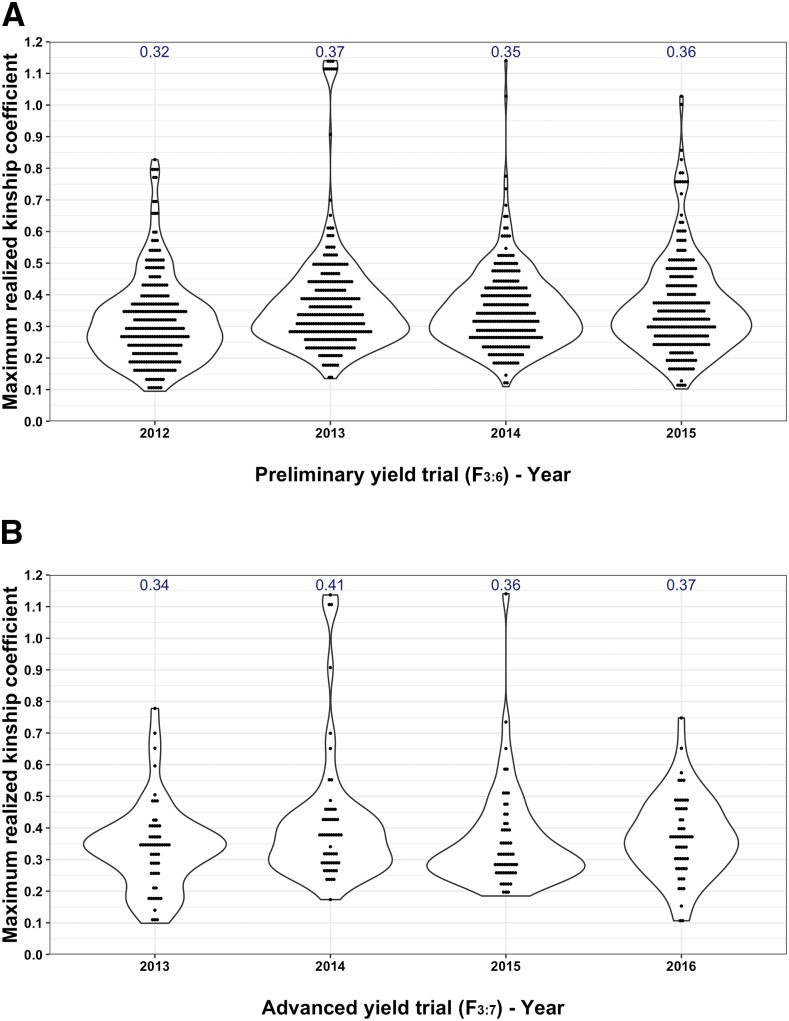
The maximum realized kinship coefficient (MRKC) between lines grown in a testing year and lines in rest of the years (training set). The MRKC was calculated as max (*U_ij_*) and *U_ij_* is the realized kinship coefficient between line *i* in the test set and line *j* in the training set. The MRKC value represents the kinship between a specific line in the test set and all lines in the training set. The kinship of each of the preliminary yield trials (PYT 2012-2015) with rest of the PYTs (training set) and kinship between lines advanced from the PYT to advanced yield trial (AYT) each year (example, AYT 2013) and lines in the training set (PYT 2013, 2014, and 2015) are shown in a and b. The average maximum realized kinship coefficient estimated across all lines grown in PYT and AYT is written on top of each of the violin plots.

The average MRKC value for the PYT grown in 2012-2015 with other PYTs ranged from 0.32 to 0.37 (Figure 1a). The PYT 2013 and 2014 contained a few lines that had a substantially higher kinship with PYT lines grown in other years (Figure 1a). The results were similar after subsetting MRKC values for the lines advanced from PYT to AYT and the average MRKC for the lines advanced each year and tested in AYT in 2013-2016 ranged from 0.34 to 0.41 (Figure 1b).

### Genomic predictions made on F_3:6_ nurseries grown in 2012-2015

**Figure 2:**
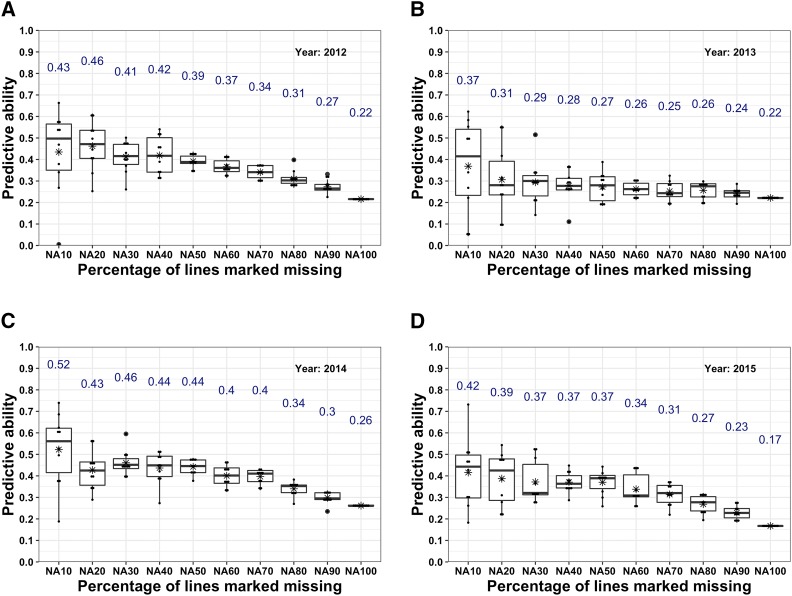
Predictive ability (PA) estimated for grain yield using various cross-validation scenarios (NA10 to NA90) and for an entire new preliminary yield trial nursery (NA100) in each of the four years, 2012 to 2015, a to d. Each of cross-validation scenarios was performed 10 times and the mean PA is written on top of each of the box plot and is also highlighted with an asterisk in the boxplots.

We predicted the performance of 10% (NA10) to 90% (NA90) of the lines grown annually in steps of 10% and repeated the random sampling of lines for the test set 10 times and estimated the average predictive abilities. In three of the four years (2012, 2014, and 2015), the average predictive abilities for NA10 ranged from 0.42 to 0.52, NA50 from 0.37 to 0.44, and NA90 from 0.23 to 0.30 (Figure 2). In 2013, the predictive abilities were slightly lower than in the other years and average predictive abilities for NA10, NA50, and NA90 scenarios were 0.37, 0.27, and 0.24 (Figure 2b). Average predictive abilities steadily decreased from NA10 to NA90 in each year. This may be due to the varying degree of kinship between training and test set resulting from increase in the number of lines in the test set from ∼27 lines in NA10 to ∼243 lines in NA90 or a decrease in the number of lines in the training set from ∼1,073 in NA10 to ∼857 in NA90 (Figure 2; Table 1). In addition, the genotype by environment interactions may impact the PA (Table S2 in File S1). We also investigated the capability to predict all lines (∼270) in a year using data from other years, the NA100 scenario. The predictive abilities for predicting an entire trial each year (2012-2015) ranged from 0.17 to 0.26 (Figure 2). The range of PA in the NA100 scenarios suggests the influence of genotype by environment interaction on PA (Table S2 in File S1).

### Genomic selection *vs.* phenotypic selection

**Figure 3:**
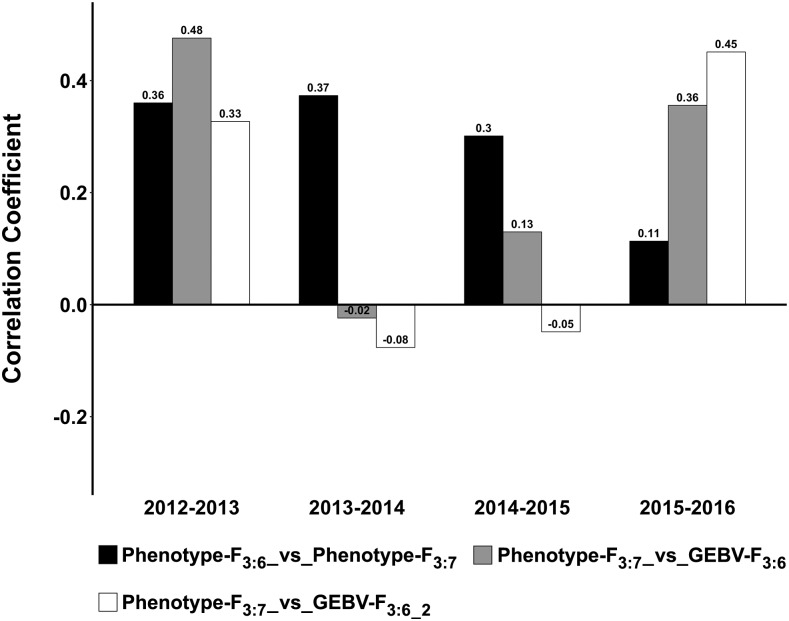
Comparison of phenotypic and genomic selection. The comparison is made using 57 lines advanced from the preliminary yield trial (F_3:6_) grown in 2012-2015 to the advanced yield trial (F_3:7_) grown in 2013-2016. The suffix “2” separated by an underscore represents scenarios where the genomic estimated breeding values of the preliminary yield trials (F_3:6_) are estimated by excluding the following (F_3:7_) year from the training set. Correlation coefficients noted on the bar plots.

The AYT nurseries grown in 2013-2016, containing 57 lines advanced primarily based on the phenotype from the PYT nurseries grown in 2012-2015, were utilized to compare phenotypic selection and GS. In 2012 and 2015, the correlation coefficient between GEBV (estimated using NA100 scenario) of the PYT nursery and BLUP of the AYT nursery was higher than the correlation coefficient between BLUP of the PYT and AYT nursery, 0.48 *vs.* 0.36 in 2012-2013 and 0.36 *vs.* 0.11 in 2015-2016 (Figure 3). When the AYT year was excluded from the training set, the correlation coefficient between GEBV of the PYT nursery and BLUP of the AYT nursery was similar or substantially higher than the correlation coefficient between BLUP of the PYT and AYT nursery, 0.36 *vs.* 0.33 in 2012-2013 and 0.45 *vs.* 0.11 in 2015-2016 (Figure 3). This suggests GS would either perform equally well or outperform phenotypic selection during 2012-2013 and 2015-2016. However, in 2014-2015, the correlation coefficient between GEBV of the PYT nursery and BLUP of the AYT nursery was nearly half (or lower than) the correlation coefficient between BLUP of the PYT and AYT nursery, 0.13 *vs.* 0.30, and -0.05 *vs.* 0.30 when the AYT year was excluded from the training set (Figure 3). And in 2013-2014, the correlation coefficient between GEBV of the PYT nursery and BLUP of the AYT nursery was significantly lower than the correlation coefficient between BLUP of the PYT and AYT nursery, -0.02 (-0.08 skipping the AYT year) *vs.* 0.37 (Figure 3). This indicates phenotypic selection would outperform GS in 2013-2014 and 2014-2015.

### Tracking selections and comparison of observed phenotypes and genomic estimated breeding values

**Figure 4:**
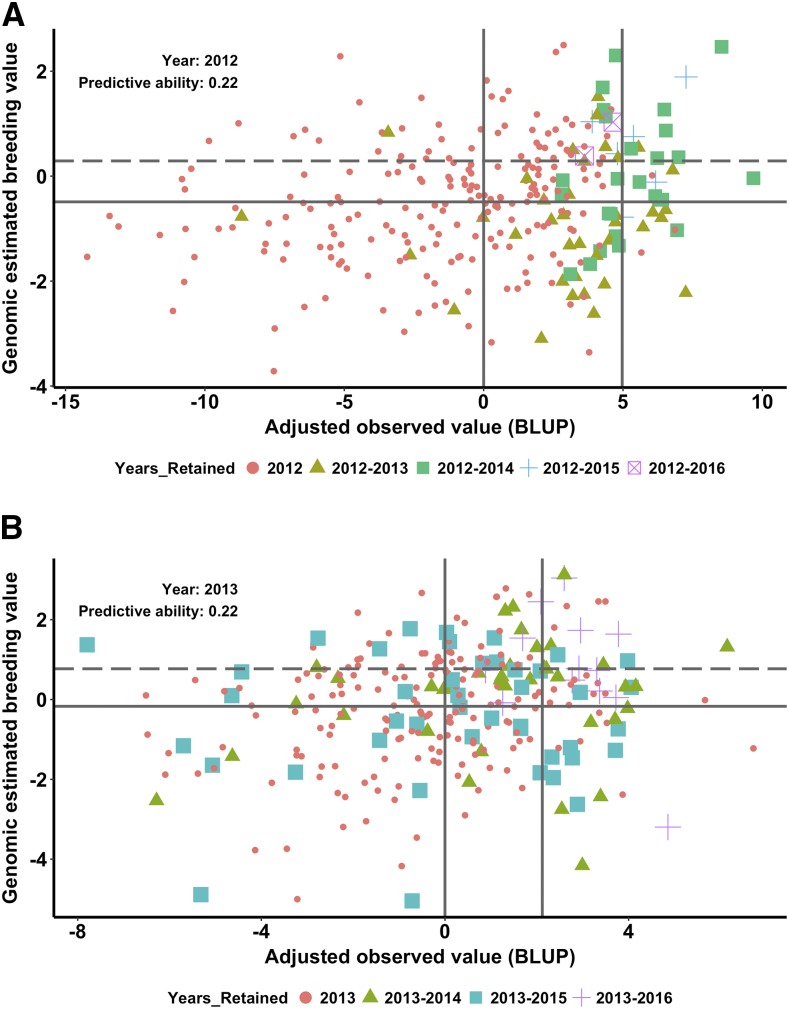
Comparison of adjusted observed grain yield values (best linear unbiased predictors, BLUPs) and genomic estimated breeding values (GEBVs) for lines tested in 2012 (a) and 2013 (b) preliminary yield trials (PYTs). The lines advanced in each year from the 2012 and 2013 PYT nursery until 2016 are highlighted with different colors and shapes. The two vertical lines represent the mean of the BLUPs of the PYT nursery and the mean of the BLUPs of the 57 PYT lines selected for advancement to the advanced yield trial nursery. The two horizontal lines indicate the mean (solid) and the 75^th^ percentile of the genomic estimated breeding values (dashed).

To examine the prospects of using GS in the PYT for making selections in the breeding program, we compared the advancements made primarily based on phenotype for five years 2012-2016 with the BLUP and GEBV values estimated on the lines in the PYT nurseries grown in 2012-2015 (Figure 4; Figure S6 in File S1). Lines with both BLUP and GEBV values above the respective means were retained for more years in the breeding program. If the lines had just the BLUP or GEBV alone above the mean but not both, then these lines were dropped in subsequent years from the breeding program (Figure 4; Figure S6 in File S1). For example, in the 2012 PYT nursery, 36 of the 71 (∼50%) lines retained for two years had the BLUP and the GEBV values above the means. Subsequently, 25 of the 34 (∼74%), 7 of the 8 (87%), and 2 of the 2 (100%) lines retained for 3, 4, and 5 years had the BLUP and the GEBV values above the means (Figure 4a). Clearly, lines with both above average GEBV and BLUP values from each year are retained for more years. The predictive abilities for the four years are in the range of 0.17 to 0.26 (Figure 4; Figure S6 in File S1). Although these prediction abilities seem low, it is interesting to note that using both GEBV and BLUP for grain yield can increase the accuracy of selections from the PYT nursery in the breeding program.

In 2017, one (NE12561) of the two lines from the 2012 PYT nursery and five (NE13434, NE13515, NE13604, NW13493, NW13570) of the 13 lines from 2013 PYT are still retained and are tested in the elite yield trails (F_3:8_ nursery; Nebraska Interstate Nursery). Three lines from 2013 PYT (NE13515, NW13493, NW13570) are also tested in the Southern Regional Performance Nursery. A few of these lines are included in the state variety testing program and they have performed well (data not shown).

**Figure 5:**
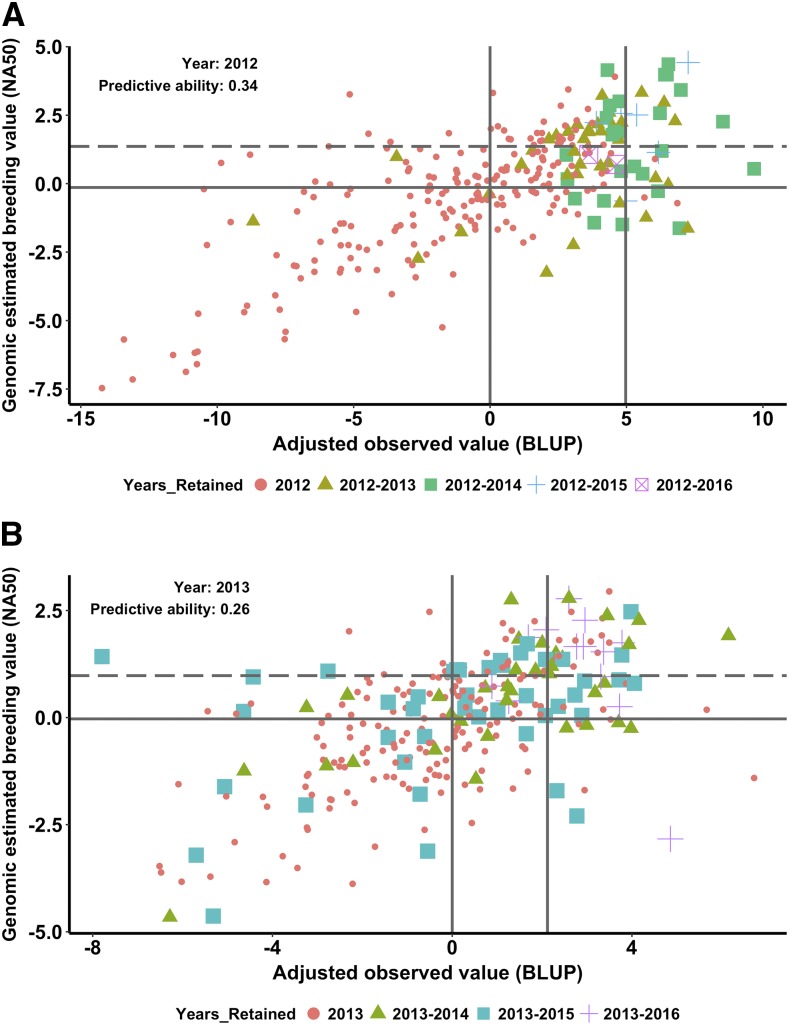
Comparison of adjusted observed grain yield (best linear unbiased predictors, BLUPs) and genomic breeding values estimated using all lines in other years and 50% of the lines randomly selected from 2012 (a) and 2013 (b) from preliminary yield trials (PYTs). The lines advanced in each year from the 2012 and 2013 PYT nursery until 2016 are highlighted with different colors and shapes. The two vertical lines represent the mean of the BLUPs of the PYT nursery and the mean of the BLUPs of the 57 PYT lines selected for advancement to the advanced yield trial nursery. The two horizontal lines indicate the mean (solid) and the 75^th^ percentile of the genomic estimated breeding values (dashed).

Next, we repeated the same analysis with the NA50 scenario of predictions in which 50% of the lines evaluated annually were added to the training dataset and predictions were made on the remaining 50% of the lines (Figure 5; Figure S7 in File S1). This analysis was performed to test the use of GEBV and BLUP to make selections when the predictive abilities are higher than in the NA100 scenario. More lines with both above average GEBV and BLUPs are retained for more years in the breeding program compared to the NA100 scenario (Figure 4). Also, lines with either their GEBV or BLUP alone higher than the respective means but not both were significantly reduced (Figure 5; Figure S7 in File S1). The predictive abilities for the four years for the NA50 scenario were higher than the NA100 scenario and were in the range of 0.26 to 0.37 (Figure 5; Figure S7 in File S1).

Experimental line NE13625 is an exception to the observation that the lines retained for more years in the breeding program have both BLUP and GEBV values about their respective means. NE13625 was the fourth highest yielding line in the 2013 PYT but had extremely low GEBV. It was advanced until 2016 and is marked with a “+” sign in the lower right quadrant in Figure 4b and Figure 5b. The line was dropped in 2016 and did not make it to the 2017 nursery. This experimental line is a relatively tall wheat, and there is a preference to retain a few tall lines that may be high yielding relative to the existing commercially available tall lines or those in the breeding program.

## Discussion

### Genotyping-by-sequencing derived genome-wide markers for genomic selection

Genotyping-by-sequencing provided thousands of high-quality SNPs after quality filtering for GP analysis. In this study, we utilized 188-plexing and obtained nearly 27,000 high-quality SNPs distributed across the genome. The number of SNPs obtained in this study using GBS after quality filtering is comparable (Dawson *et al.* 2013; Poland *et al.* 2012a) or higher than the previously published articles on genomic predictions in wheat (Battenfield *et al.* 2016; Rutkoski *et al.* 2014). SNP calling accuracy was high (∼95%) when tested using biological replicates with small samples of F_3:6_ lines and multiple check cultivars. Also, the percentage of SNP calling accuracy observed in this study using GBS is similar to other platforms such as RNA-Seq (Belamkar *et al.* 2016) and targeted resequencing of the wheat exome (Allen *et al.* 2013). The GBS is a cost-efficient, reliable, and powerful technique for rapidly genotyping breeding germplasm with genome-wide markers. In addition, a cost-effective strategy for a self-pollinated crop such as wheat would be to genotype lines from a breeding program nursery with a manageable number of lines and then use the same markers for lines that are advanced. For example, we are presently genotyping F_3:5_ nursery in the University of Nebraska breeding program that contains nearly ∼2,000 lines annually and using the same marker data for lines advanced to F_3:6_ and F_3:7_ nurseries. The error introduced due to the minor change in average heterozygosity with each generation is acceptable compared to the cost of re-genotyping lines advanced to the subsequent nurseries. This approach has also been demonstrated recently in a different wheat breeding program (Michel *et al.* 2017).

### Genomic predictions made on preliminary yield trial nursery grown in 2012-2015

Genomic predictions made in each year (2012-2015) indicate predictive abilities are high for NA10, modest for NA50, and low for NA100. Also, the variability in the predictive abilities is higher from NA10 to NA40 compared to NA50 to NA90. Higher predictive abilities obtained on a smaller number of lines (NA10) and relatively lower predictive abilities on a larger number of lines (NA90) in the test set is probably due to the sample size differences and kinship between lines in the training and test sets. Predictive ability is a function of heritability of the trait, the size of the training population, and an effective number of chromosome segments affecting the trait (Daetwyler *et al.* 2008). Training and test population sizes affecting predictive abilities have also been demonstrated in previous studies on various species (Akdemir *et al.* 2015; Asoro *et al.* 2011; Cericola *et al.* 2017; Tayeh *et al.* 2015). The high variation in the predictive abilities with fewer lines (NA10-NA40) in the test set is probably due to a varying degree of kinship between lines in the test and training set (Weng *et al.* 2016). Recently, He *et al.* (2017) showed that kinship is more important than genetic architecture of trait for determining the estimates of GP on grain yield in wheat. The PA of new lines in a new year using lines from other years (NA100 scenario) was low (∼0.22). Besides kinship, genotype by environment interaction can be responsible for these low values. Although same lines were not tested across years in the PYT, checks were grown in multiple years. Ranking of these checks indicated the influence of genotype by environment interactions on grain yield in different years (Table S2 in File S1). For instance, ‘Goodstreak’ was lower yielding than ‘Camelot’ in all years except in 2015 when it yielded significantly higher. Similarly, Camelot and ‘Freeman’ yielded nearly the same in 2014 whereas in 2015 Freeman yielded higher than Camelot. In summary, these results with the current training dataset generated (containing four PYT nurseries) in the University of Nebraska wheat breeding program indicate that we can predict the yield of nearly 50% of the lines in the PYT nursery with reasonable (0.37 to 0.44) predictive abilities. We believe it may be possible to reduce costs by phenotyping only a subset of lines, especially for hard to measure traits (with similar genetic architecture and heritability as yield), and also recover information for a subset of lines lost during extreme weather events such as a hailstorm or other abiotic and biotic stresses. However, predicting the yield of all lines in the PYT nursery in a new year (forward breeding) and skipping the PYT is not possible with the current selection intensity of advancing 57 of the 270 lines to the AYT.

### Evaluation of genomic selection and phenotypic selection

The comparison of GS and phenotypic selection for making advancements from the PYT to the AYT nursery indicated GS did better than the phenotypic selection in 2012 and 2015, and in 2013 and 2014 phenotypic selection outperformed GS. When the AYT year was excluded from the training set (for example, predicting PYT 2012 using PYT 2014 and 2015 and skipping the PYT in the AYT year 2013 in the training set), GS was similar to phenotypic selection in 2012 (which makes GS superior because if both GS and phenotypic selection have the same efficiency, we can skip phenotyping), GS did better than phenotypic selection in 2015, and phenotypic selection outperformed GS in 2013 and 2014. The lower PA of GS compared to phenotypic selection after excluding the AYT year in the training set is likely either due to the smaller training set or the fact that the AYT year was not observed in the training set. Overall, GS did better than phenotypic selection in 2012 and 2015, and the phenotype selection was better than GS in 2013 and 2014.

In 2012, Nebraska experienced severe drought (Baenziger *et al.* 2012), and in 2015 it received unusually high amounts of rain especially in May, which coincides with the initiation of the flowering of the NE lines (Baenziger *et al.* 2015). The 2015 crop year was the third wettest year since the national records began in 1895, and May 2015 was the wettest month of any month on record (NOAA 2016). The high amounts of rain resulted in unusually severe disease pressure in 2015, primarily due to stripe rust (causal organism *Puccinia striiformis* f. sp. *tritici*) and *Fusarium* head blight (causal organism *Fusarium graminearum Schwabe* [teleomorph *Gibberella zeae* (Schwein.) Petch]) (Baenziger *et al.* 2015). The 2014 crop year did not witness any extreme weather events such as drought, flooding, or hailstorms and was a relatively normal year (Baenziger *et al.* 2014). In both the extreme weather years (2012 and 2015), GS was comparable or better than phenotypic selections, whereas in a normal year (2014) the GS PA was nearly 50% lower than the phenotypic selection. It is interesting to note that GS did better than phenotypic selection while the predictive abilities for the nurseries grown in 2012 and 2015 were 0.22 and 0.17. This suggests predictive abilities alone are most likely not a true indicator of the success of genomic predictions, and the low values obtained may be due to the quality of the underlying phenotype data during extreme weather years. Based on these results in the PYT nursery, GS alone is effective for making selections when extreme weather events occur during the growing season.

The results obtained in 2013 were quite different from all other years. The GS abilities were quite low compared to phenotypic selection. Also, the predictive abilities obtained for NA10 to NA90 were relatively lower. This is despite the fact that the kinship of lines grown in PYT 2013 with lines grown in PYTs in other years was similar to the kinship among other PYTs. In addition to kinship, we specifically looked at the clustering of the lines advanced from PYT 2013 to AYT 2014 with the rest of the PYT lines (Figure S5 in File S1). A plot of PC1 *vs.* PC2 and a parallel coordinate plot with five PCs did not suggest that these lines were different from the rest (Figure S5 in File S1). This result is expected because each experimental line in the University of Nebraska wheat breeding program is derived from a cross made with one or more lines adapted to the Nebraska region, and thus strong population structure is not expected among the breeding program lines. The possible reasons that may have affected the PA values are: (1) the phenotype of lines grown in 2013 was influenced by the quality of seeds harvested in 2012 coupled with the soil and field conditions affected following severe drought stress in 2012 (Baenziger *et al.* 2013); (2) the broad-sense heritability values of PYT 2013 and AYT 2014 are lowest compared to PYTs and AYTs in other years (3) the selections performed in 2012 OYT nursery to advance lines to PYT nursery grown in 2013 would have been difficult to make accurately due to the drought stress; and (4) the GBS data of ∼92 lines grown in 2013 was of relatively lower quality (but still acceptable for including it in the analysis based on per base sequence average quality scores >20 along the length of the GBS tags). The most common reasons for lower quality scores is a degradation of quality over the duration of long sequencing runs or a problem with the sequencing run such as bubbles passing through a flowcell (http://www.bioinformatics.babraham.ac.uk/projects/fastqc/).

### Genomic selection is promising for increasing selection accuracy

In the PYT, lines with both above average BLUP and GEBV values were retained for more years compared to lines with either just GEBV or BLUP alone. A limitation of using phenotype data alone for making selections is that they are based on the performance of lines only in the growing year. But, the expectation is that the selected line performs well in multiple years with potentially varying environmental conditions. The GEBV estimates based on lines grown in multiple years and multiple environmental conditions leveraged information across years and environments and overcomes the limitation of phenotype-based single-year selection. Thus, using both BLUP and GEBV independently and selecting lines that are having both their GEBV and BLUP above the respective means and excluding lines with just the GEBV or BLUP alone above the mean provided an increased opportunity to select lines likely to perform well across environments and years compared to lines selected based on the phenotype alone in one year.

At present, with the current training dataset, the PA is approximately 0.22 for predicting all lines in the PYT nursery in a new year. Hence, with the current selection intensity (selection of 57 of the 270 lines; Figure S3 in File S1) for the subsequent nursery (AYT nursery), we can make highly accurate selections by using both phenotype and GEBV values. Another possibility would be to lower the selection intensity by selecting all lines above the average GEBV for the subsequent nursery. This would allow skipping the PYT, but the downside is the cost of many more lines advanced to the elite trials replicated and grown in many locations, which makes this approach practically challenging and potentially cost ineffective in a breeding program.

The percentage of lines with above average GEBV and BLUP retained for more years in the breeding program increased when 50% of the lines tested in the prediction year are added to the training dataset and predictions are made on the remaining 50% of the lines. The predictive abilities for this NA50 scenario are higher than the NA100. In 2014, the predictive ability was greater than 0.37, and very few lines were in the quadrant with above average GEBV and lower than average BLUP. Our data suggest that growing only a subset of PYT and combining the information from previous years will effectively utilize GEBV for making selections for yield. Also, these results indicate when the predictive ability surpasses the average predictive abilities observed in the NA50 scenario (∼0.40), we may be able to use just GEBV alone for making selections with the current selection intensity for the subsequent nursery and skip the PYT. We have more than ∼1,000 g of seed for most of the lines in the OYT (F_3:5_), and thus enough seed is available to skip the PYT and test lines in the AYT. In addition, we now genotype the lines in OYT and the marker data will be available for lines selected for PYT prior to testing them in the field.

In summary, our recommendation while the predictive abilities for forward breeding are lower (∼0.20), is to either use both the GEBV and BLUP values to make better selections or grow only a selected subset of lines (especially lines that are difficult to predict) advanced to the PYT nursery and use GS to predict the rest and make selections in the PYT nursery. The subset of lines whose phenotype is difficult to predict can be determined by using the reliability criterion recently described by Yu *et al.* (2016) and the training set can be updated annually as described by Neyhart *et al.* (2017).

### Current use of genomic selection in the University of Nebraska wheat breeding program

The results obtained in this study are encouraging, and this has motivated implementation of GS in the breeding program for grain yield starting in 2016. Here, we describe three examples of the current use of GS. First, in 2016, we used both GEBV and BLUP to make more accurate selections from the PYT nursery. Preliminary observations indicate in 2017 more lines with both above average GEBV and BLUP were advanced to 2018 and lines with either just above average GEBV or BLUP alone were dropped. Second, lines with both above average GEBVs and BLUPs were shortlisted as top performing lines in the PYT nursery and used as parental lines for the subsequent year’s crossing block. Recycling elite lines sooner saved one to two years in the breeding cycle. Third, hailstorms damaged more than half the OYT nursery (F_3:5_) containing nearly 2,000 lines mostly grown at one location in 2016. We used a training dataset comprising PYT nurseries plus 50% of the lines from the OYT nursery that were less hail damaged to predict the phenotype of the remaining 50% of the lines in the OYT nursery damaged due to hail and recovered lines that had the potential for high-yielding based on GS and advanced these to the PYT nursery for harvest in 2017.

Overall, integration of GS for grain yield in the University of Nebraska wheat breeding program is positioned to successfully improve the efficiency of the breeding program. We currently are testing PAs of other traits and investigating approaches to improve the use of GS in the breeding program.
